# First report of *Pratylenchus parazeae* (Nematoda: Pratylenchidae) associated with rice in Vietnam

**DOI:** 10.2478/helm-2025-0024

**Published:** 2025-11-26

**Authors:** T. D. Nguyen, T. M. L. Le, H. T. Nguyen, H. A. Le, Q. P. Trinh

**Affiliations:** 1 Institute of Biology, Vietnam Academy of Sciences and Technology, 18 Hoang Quoc Viet, Cau Giay 100000 Hanoi, Vietnam; 2 Graduate University of Science and Technology, Vietnam Academy of Sciences and Technology, 18 Hoang Quoc Viet, Cau Giay, 100000 Hanoi, Vietnam

**Keywords:** *Oryza sativa*, root-lesion nematode, morphology, molecular identification, 28S D2-D3 rDNA, 18S rDNA, phylogenetic analysis, plant-parasitic nematodes

## Abstract

Members of the genus *Pratylenchus*, commonly known as root-lesion nematodes, rank among the most economically important plant-parasitic nematodes worldwide due to their broad host range, wide distribution, and ability to cause significant yield losses in major crops, including rice, maize, and sugarcane. They invade root tissues, creating lesions that impair water and nutrient uptake, reduce plant growth, and increase susceptibility to secondary infections. *Pratylenchus parazeae*, a root-lesion nematode previously known from sugarcane and maize in China, is reported for the first time in Vietnam, associated with rice (*Oryza sativa* L.) in Vinh Phuc Province. This study provides an integrative identification of the species based on detailed morphological features and molecular characterization using 18S rDNA and D2-D3 segments of the 28S rDNA. Female specimens exhibited diagnostic characteristics consistent with *P. parazeae*. No males were observed. The D2-D3 and 18S rDNA sequences showed 98.7 – 99.0 % identity with previously described *P. parazeae* sequences, and phylogenetic analysis placed the Vietnamese population within a well-supported clade alongside known *P. parazeae* isolates, distinct from other closely related *Pratylenchus* species. This first record of *P. parazeae* on rice in Vietnam expands the known host range and geographic distribution. The findings underscore the need for targeted surveillance and management strategies to mitigate the potential threat of *P. parazeae* to rice production in Southeast Asia.

## Introduction

Rice (*Oryza sativa* L.) is one of the most vital staple crops globally (Mohidem *et al*., 2022). In Asia-Pacific, including Vietnam, rice plays a central role in food security and national agricultural productivity, with over 90 % of global rice production and consumption concentrated in this area (Tan & Norhaizan, 2020). However, rice production faces serious threats from numerous biotic stressors, among which plant-parasitic nematodes are of significant concern. These microscopic parasites, especially those belonging to the genus *Pratylenchus* (commonly referred to as root-lesion nematodes), can cause significant yield losses in rice under severe infestations (Bridge *et al*., 2005; Sikora *et al*., 2018).

The genus *Pratylenchus* comprises migratory root endoparasitic species (Castillo & Vovlas, 2007). Species within this genus are globally distributed and ranked among the most economically damaging plant-parasitic nematodes (Castillo & Vovlas, 2007; Jones *et al*., 2013). They invade root tissues, producing necrotic lesions that impair plant growth and reduce crop yields. Currently, over 100 *Pratylenchus* species have been described, each exhibiting different host ranges and pathogenic capacities (Geraert, 2013; Nguyen *et al*., 2019). In total, 14 *Pratylenchus* species have been recorded from Vietnam, parasitizing many key crops such as coffee, black pepper, pineapple, sugarcane, maize, and carrots (Nguyen *et al*., 2023). Among these, *P. zeae* and *P. coffeae* have been previously reported to be associated with rice in Vietnam (Nguyen *et al*., 2023; Nguyen & Nguyen, 2000).

*Pratylenchus parazeae* was initially reported from sugarcane (*Saccharum sinensis*) in Guangxi, China (Wang *et al*., 2015), and has also been detected on maize in China (Wu *et al*., 2019). While *P. parazeae* has been noted as a damaging nematode on sugarcane in China, its presence on other crops and in other countries has not been well documented. This study reports, for the first time, the occurrence of *Pratylenchus parazeae* associated with rice in Vietnam. The identification was based on detailed morphological and molecular analyses of the 18S and D2-D3 segments of the 28S region. This finding adds to the known host range and geographical distribution of *P. parazeae* and provides important implications for nematode management in rice cultivation in Vietnam and Southeast Asia.

## Materials and Methods

### Sampling and nematode extraction

Soil and root samples were randomly collected from rice fields in Vinh Phuc, Vietnam (21°20’32”N; 105°25’25”). Each sample was placed in an individual nylon bag and transported to the laboratory for nematode extraction. Nematodes from soil were extracted using the decanting and sieving technique following the protocol of Nguyen (2003). For root samples, segments approximately 0.5 cm in length of roots were prepared and subjected to nematode extraction using a modified Baermann tray method (Whitehead & Hemming, 1965).

### Morphological characterisation

Extracted nematodes were heat-killed in water maintained at 60 – 70 °C for 30 seconds, then fixed in TAF solution (a mixture of 8 % formalin and 2 % triethanolamine in distilled water) for 4 – 5 days, according to the procedure of Nguyen (2003). Subsequently, nematodes were dehydrated following a modified Seinhorst method (Seinhorst, 1959). Specimens were first transferred to Seinhorst I solution (20 % ethanol + 1 % glycerol) and placed in a small open staining dish inside a sealed container with 96 % ethanol (approx. 1/10 volume). The container was incubated overnight at 38°C. The next day, the dish was removed and placed in an oven at 38°C, partially covered. Every 60 minutes, approximately 50 μL of Seinhorst II solution (pure glycerol) was added (four times). After overnight incubation, the ethanol had evaporated, leaving nematodes in pure glycerol. Nematodes in glycerol were then mounted on permanent glass slides for microscopic examination. Morphometric data and photomicrographs were obtained using a Carl Zeiss Axio Lab.A1 compound microscope.

### Molecular characterisation

For molecular characterization, individual live nematodes were cut into small fragments to enhance DNA extraction efficiency. The tissue fragments were transferred into PCR tubes containing 20 μl of worm lysis buffer (50 mM KCl; 10 mM Tris-HCl, pH 8.3; 2.5 mM MgCl_2_; 0.45 % NP-40 (Tergitol Sigma); and 0.45 % Tween-20), followed by the addition of 2 μl of proteinase K (1.2 mg/ml). The tubes were incubated in a thermal cycler at 65 °C for 1 hour, then heated to 95 °C for 10 minutes to inactivate the proteinase K. After incubation, samples were centrifuged at 8000 rpm for 1 minute. The extracted DNA was stored at –20 °C until further use.

PCR amplification of the D2-D3 segment of the 28S rDNA gene was performed using primers D2A (5'–ACAAGTACCGTGGGGAAAGTTG–3') and D3B (5'–TCGGAAGGAACCAGCTACTA–3') (Nunn, 1992), while the 18S rDNA region was amplified using primers MN18F (5'–CGCGAATRGCTCATTACAACAGC–3') and Nem_18S_R (5'–GGGCGGTATCTGATCGCC–3') (Floyd *et al*., 2005). Each PCR reaction (25 μl total volume) contained 12.5 μl of 2X Hot Start Green PCR Master Mix (Promega, USA), 1 μl of each primer (10 μM), 3 μl of DNA template, and sterile Milli-Q water to adjust the final volume. PCR was carried out using a SimpliAmp Thermal Cycler (Thermo Fisher Scientific) with the following program: initial denaturation at 95 °C for 4 minutes; 40 cycles of denaturation at 95 °C for 30 seconds, annealing at 54 °C for 30 seconds, and extension at 72 °C for 60 seconds; followed by a final extension at 72 °C for 5 minutes. PCR products were visualized by electrophoresis on a 1 % agarose gel stained with GelRed and observed under UV light. Successful amplicons were purified using ExoSAP-IT™ (Thermo Fisher Scientific) and sent for sequencing to Apical Scientific (Selangor, Malaysia). Sequence identity was assessed using BLAST searches against the NCBI database. Homologous sequences were aligned using ClustalW, and phylogenetic trees were constructed using MEGA 12 (Kumar *et al*., 2016) based on the best-fit substitution model selected according to the Bayesian Information Criterion.

## Ethical Approval and/or Informed Consent

Not applicable.

## Results

### Pratylenchus parazeae Wang, Zhuo, Ye & Liao, 2015

*Pratylenchus parazeae* was detected in 66.7 % of the examined samples, with a mean density of 88 individuals per 250 g of soil in the positive samples.

(Fig. 1)

**Fig 1.**
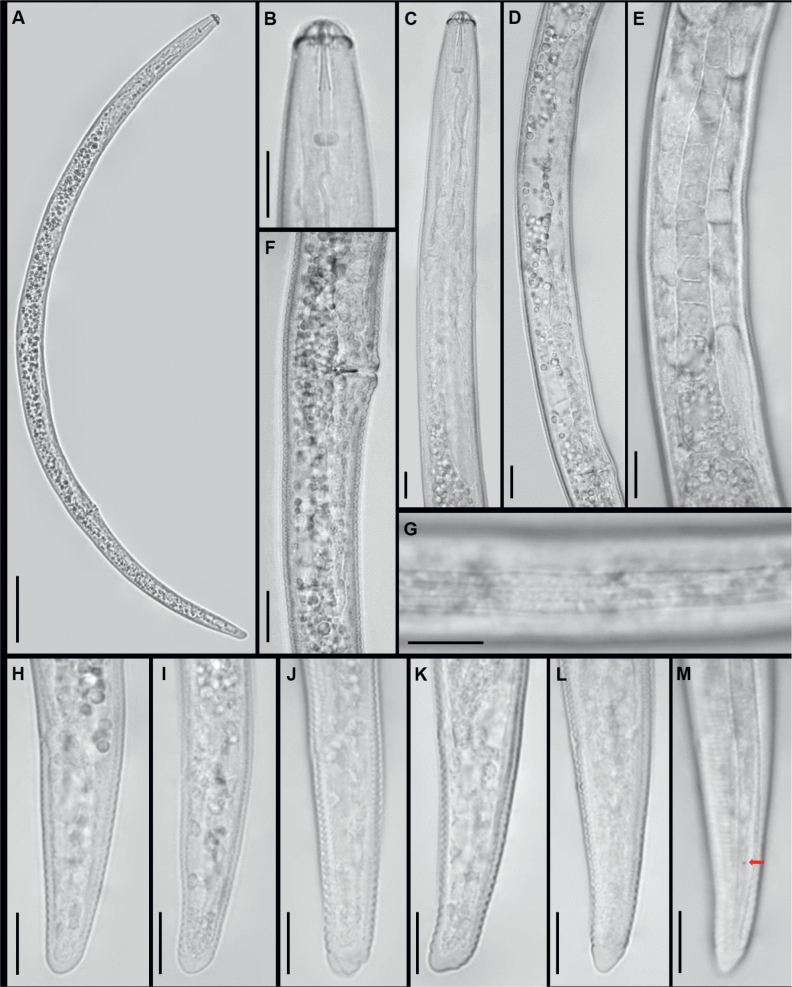
Females of *Pratylenchus parazeae* from Vietnam under the light microscope. A: entire body; B-C: anterior region; D: vulval region; E: ovary; F: post-vulval uterine sac; G: lateral field at midbody; H-M: tail region. (Arrows point to phasmids; scale bars: A=50 μm; B-M=10μm).

### Measurements

Measurements are presented in Table 1.

**Table 1.** Measurements of *Pratylenchusparazeae* from Vietnam and China. All measurements are in μm (except for ratio) and in the form: mean±s.d. (range)

Characters	Vinh Phuc, Vietnam	Baise city, Guangxi, China	Laibin city, Guangxi, China	Hechi city, Guangxi, China	Laibin city, Guangxi, China
	This study	Wang *et al*., 2015	Wang *et al*., 2015	Wang *et al*., 2015	Wu *et al*., 2019
**n**	26	20	20	20	12
**L**	576±33 (519-638)	588±38 (528–683)	628±43 (532–705)	649±26 (600–697)	605 (486-686)
**Lip height**	3±0.3 (2.6-3.3)	2.9±0.2 (2.5–3.2)	2.5±0.2 (2.2–3.0)	2.8±0.2 (2.5–3.4)	-
**Lip width**	7.6±0.3 (7.1-8.6)	8.6±0.4 (8–9.2)	8.6±0.3 (8.0–9.1)	8.6±0.3 (7.8–9.1)	-
**Stylet length**	17.4±0.3 (16.9-18.2)	17.3±0.4 (16.7–18.1)	17.7±0.4 (17.0–18.5)	18.3±0.3 (17.7–19.2)	18.3 (16.5-19.2)
**Shaft length**	8.9±0.4 (8.5-9.8)	9.1±0.3 (8.3–9.7)	9.5±0.3 (8.6–10.0)	9.8±0.2 (9.5–10.5)	-
**Knob height**	2.2±0.2 (1.8-2.5)	2.3±0.2 (2.0–2.5)	2.3±0.2 (2.0–2.7)	2.4±0.1 (2.1–2.6)	-
**Knob width**	4±0.3 (3.6-4.7)	4.1±0.2 (3.9–4.5)	4.3±0.3 (3.9–4.7)	4.2±0.2 (3.8–4.6)	-
**DGO**	3.6±0.3 (3-4)	3.1±0.3 (2.5–3.7)	3.0±0.3 (2.6–3.4)	3.1±0.3 (2.6–3.6)	-
**Anterior end to center of median bulb**	59±3 (52-66)	60±4 (52–67)	56±3 (52–62)	60±2 (55–65)	-
**Median bulb length**	12.9±0.9 (11.3-14.8)	13.7±0.8 (12.8–15.3)	13.5±0.6 (12.6–14.7)	14.7±0.7 (13.9–15.8)	-
**Median bulb width**	10±0.8 (8.6-11.7)	10.4±0.9 (9.1–11.8)	10.8±0.4 (10–11.3)	11.1±0.4 — (10.8–11.9)	-
**Anterior end to nerve ring**	72±3 (67-78)	-	-	-	-
**Anterior end to excretory pore**	87±5 (78-97)	84±4 (76–90)	94±6 (82–103)	93±5 (78–99)	-
**Anterior end to cardia**	96±5 (86-105)	93±5 (84–103)	90±4 (85–99)	93±4 (86–102)	-
**Anterior end to end of pharyngeal gland**	137±7 (122-148)	130±6 (117–139)	140±9 (123–158)	137±8 (125–158)	-
**Pharyngeal overlapping**	41±4 (33-47)	38±4 (29–44)	50±9 (31–66)	44±7 (34–66)	-
**Cuticle annuli width**	1.3±0.2 (1-1.7)	-	-	-	-
**Max. body diam**.	22±3 (17.7-27)	24±2 (19.3–27)	21±1.3 (18.7–24)	24±2 (21–26)	28 (24-33)
**Vulval body diam**.	20±2 (16.7-24)	22±1 (20–24)	21±1 (18.2–23)	23±2 (20–26)	-
**Anterior genital tract length**	159±26 (121-218)	177±38 (118–263)	145±20 (115–204)	165±27 (133–243)	-
**Post-uterine sac**	43±5 (34-50)	41±6 (36–53)	40±4 (33–49)	50±6 (42–61)	-
**Vulva to anus distance**	118±14 (93-147)	121±11 (107–152)	130±12 (106–151)	152±12 (134–176)	-
**Anal body diam**.	12.6±1.2 (10.7-16.1)	14.1±1.4 (12.2–16.2)	14.1±0.7 (13.0–15.3)	15.3±0.7 (13.7–16.1)	-
**Tail length**	36±3 (32-41.9)	37±3 (32–42)	38±2 (33–42)	40±3 (35–44)	9.0 (8.1-9.2)
**No. of tail annuli**	27±3 (23-34)	25±2 (22–28)	29±2 (25–33)	32±3 (25–36)	-
**Lateral field width**	6.4±1 (5.3-9.2)	6±0.6 (5.1–7.1)	6.0±0.6 (5.2–7.3)	6.8±1.0 (5.7–7.6)	-
**Phasmid from tail terminus**	17.4±2.1 (15.2-19.9)	17.7±2.1 (15.2–21)	18.9±2.4 (16.2–24)	21±1.2 (18.9–24)	-
**V%**	73±2 (70-75)	73±1 (71–75)	73±1 (72–74)	71±1 (69–73)	72 (71-74)
**a**	26±3 (22-30)	25±2 (22–28)	30±2 (26–34)	27±2 (24–30)	26 (23-29)
**b**	6±0.3 (5.5-6.5)	6.4±0.5 (5.6–7.7)	7.0±0.5 (6.2–7.9)	7.0±0.4 (6.1–7.7)	6.3 (5.5-7.5)
**b'**	4.2±0.2 (3.8-4.5)	4.5±0.3 (4.1–5.2)	4.5±0.3 (3.7–5.2)	4.7±0.3 (4.1–5.3)	-
**c**	16.1±0.9 (14.7-17.7)	15.9±1.1 (13.5–17.3)	16.8±1.1 (14.6–18.6)	16.5±1.1 (15.3–19.6)	16.7 (13.1-20.0)
**c'**	2.9±0.3 (2.4-3.3)	2.6±0.2 (2.3–3.1)	2.7±0.2 (2.3–3.0)	2.6±0.2 (2.3–3.0)	-
**E.P. (%)**	15.2±0.9 (13.2-16.3)	14.3±0.8 (12.9–15.6)	15.0±0.5 (14.0–16.1)	14.3±0.7 (12.4–15.5)	-

### Morphological characterisation

*Female*. Body straight or ventrally curved upon heat fixation (Fig. 1A). Midbody cuticle annuli measuring 1.0 – 1.7 μm in width. Lip region with three annuli, continuous with body contour. Stylet robust, with a conical portion equal to or shorter than the shaft, constituting 45 – 50 % of the total stylet length (Fig. 1B). Stylet base well-developed and rounded (Fig. 1B). Dorsal esophageal gland orifice located 3.0 – 4.0 μm posterior to stylet base (Fig. 1B). Median bulb prominent, ovoid (Fig. 1C). Isthmus narrow, encircled anteriorly by the nerve ring (Fig. 1C). Pharyngeal glands overlapping the intestine ventrally, containing three nuclei (Fig. 1C). Secretory-excretory pore situated at or anterior to the esophago-in-testinal junction (Fig. 1C). Hemizonid located immediately anterior to the excretory pore, spanning one annulus. Reproductive system monodelphic-prodelphic, extending anteriorly; ovary with oocytes arranged in a single row (Fig. 1E). Spermatheca reduced, ovoid or spherical without sperm (Fig. 1D). Vulva slightly protruding (Fig. 1F). Post-vulval uterine sac measuring one-third to two-fifths of the distance from vulva to anus (Fig. 1F). Phasmids pore-like, located at 41 – 52 % of tail length (Fig. 1M). Tail nearly cylindrical, tapering towards the terminus, with 23 – 34 annuli (Fig.1H–L). Lateral field with four incisures, occupying approximately one-third of body diameter (Fig. 1G).

*Male*. Not found

### Molecular characterisation

D2-D3 segment of the 28S rDNA Region

Two D2-D3 sequences of the 28S rDNA region from *Pratylenchus parazeae* populations in Vietnam (accession numbers: PV355046 and PV355047) were 791 base pairs in length. These sequences exhibited 98.7 – 99.0 % identity with previously published sequences of *P. parazeae* in GenBank (KY424325, KF765433, KP903441, KY424323, KY424327, KP903445, KF765435). Intraspecific variation among *P. parazeae* sequences ranged from 0.3 % to 1.4 %. In contrast, interspecific comparisons showed a divergence of 11.3 – 12.0 % (with 7 – 18 gaps) from *P. bhatti* (JN244269, JN244270), and 65 – 70 nucleotide differences (with 10 – 17 gaps) from *P. delattrei* (JX261948, JX261949). Comparisons with *P. zeae* (OQ630471, KY424269, JN020929) revealed 72 – 95 variable sites, corresponding to a sequence divergence of 11.4 – 11.7 %, with 8 – 17 gaps.

**Fig 2.**
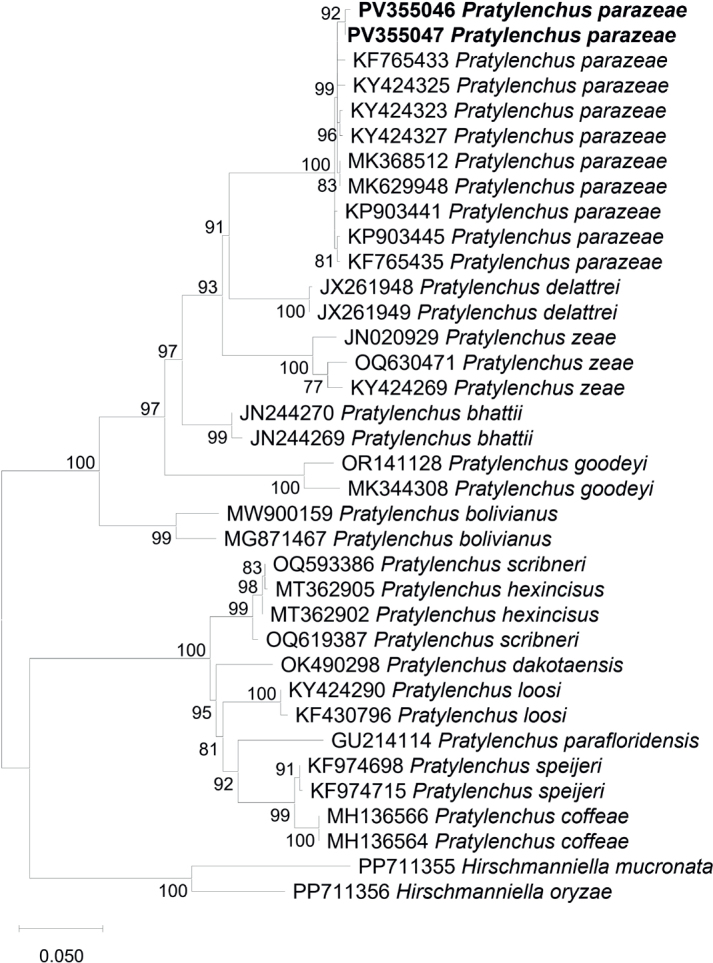
Phylogenetic relationships of *Pratylenchus parazeae* and related species based on the D2-D3 segment of 28S rDNA region, inferred using the Maximum Likelihood (ML) method with the K2+G+I model (BIC = 9596.839, AICc = 8978.033; lnL = –4412.786; +I = 0.40; +G = 1.23; R = 2.19; base frequencies: A = 0.25, T = 0.25, C = 0.25, G = 0.25). Numbers on branches represent bootstrap values from 1,000 replicates. Sequences generated in this study are indicated in bold.

The phylogenetic tree based on the D2-D3 expansion region clearly resolved seven distinct clades (Fig. 2). The *P. parazeae* sequences obtained in this study were clustered with high support alongside previously published *P. parazeae* sequences, indicating strong species-level congruence. The sister relationship between Clade I (*P. parazeae*) and Clade II (*P. delattrei*) was supported by a bootstrap value of 91 %.

### 18S rDNA Region

**Fig 3.**
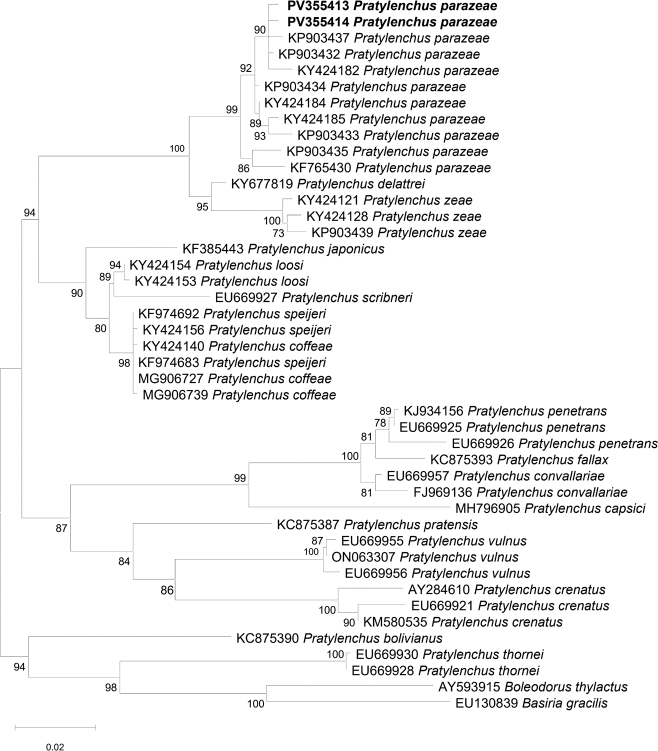
Phylogenetic relationships of *Pratylenchus parazeae* and other *Pratylenchus* species based on the 18S rDNA region, inferred using the Maximum Likelihood (ML) method with the K2+G+I model (BIC = 10340.526, AICc = 9590.047; lnL = -4706.815; +I = 0.53; +G = 0.85; R = 1.79; base frequencies: A = 0.25, T = 0.25, C = 0.25, G = 0.25). Bootstrap values from 1,000 replicates are shown on branches. Sequences from this study are highlighted in bold.

18S rDNA sequences of *P. parazeae* from Vietnam were also obtained (accession numbers: PV355413 and PV355414), showing 99.0 – 100 % identity with other *P. parazeae* sequences available in GenBank (KP903437, KP903432, KY424182, KY424184, KP903435, KP903434). Intraspecific variation within *P. parazeae* based on 18S sequences ranged from 0.3 % to 1.6 % (3 – 17 bp). Interspecific variation between *P. parazeae* and *P. zeae* ranged from 4.5 % to 5.0 % (36 – 47 bp) with 3 – 5 insertion/deletion gaps. In comparison, *P. zeae* isolates showed a lower level of intraspecific variation, between 0.7 % and 0.8 % (6 – 8 bp). The phylogenetic tree based on partial 18S rDNA sequences (Fig. 3) placed *P. parazeae* as a sister taxon to a clade consisting of *P. delattrei* and *P. zeae* with 100 % bootstrap support, and both species formed a strongly supported monophyletic group.

## Discussion

The present study constitutes the first confirmed report of *Pratylenchus parazeae* associated with rice (*Oryza sativa*) in Vietnam, expanding both the known geographical distribution and host range of this economically significant root-lesion nematode. The integration of detailed morphological analysis and robust molecular phylogenetics (D2-D3 segment of 28S rDNA and 18S rDNA) provides a strong basis for accurate species delimitation and identity confirmation, particularly given the historical taxonomic complexities within the *Pratylenchus* genus (De Luca *et al*., 2012; Wang *et al*., 2015). Morphologically, the Vietnamese population of *P. parazeae* exhibited diagnostic characters that were consistent with the original description by Wang *et al*. (2015), including a well-developed stylet with rounded knobs, a ventrally overlapping pharyngeal gland lobe, a monodelphic-prodelphic reproductive system, and a tail with 23 – 34 annuli. Notably, the absence of males in the population aligns with previous reports suggesting that *P. parazeae* may exhibit facultative parthenogenesis, a reproductive trait commonly observed in some *Pratylenchus* species under specific ecological pressures (Castillo & Vovlas, 2007). However, species-level identification within *Pratylenchus* based solely on morphology remains inherently challenging due to overlapping morphometrics and intraspecific plasticity with the presence of cryptic species (De Luca *et al*., 2012). This underscores the necessity of combining traditional diagnostic approaches with molecular tools, as adopted in the current study.

The D2-D3 segment of the 28S rDNA has proven to be a valuable molecular marker for delineating species boundaries within *Pratylenchus*, offering sufficient interspecific resolution (Subbotin *et al*., 2008). The Vietnamese sequences of *P. parazeae* showed 98.7 – 99.0 % similarity with reference sequences from sugarcane and maize populations in China, confirming their conspecific status. The apparent phylogenetic clustering of Vietnamese isolates together with other sequences of *P. parazeae* from GenBank, with strong bootstrap support, indicates no significant divergence from previously known populations, suggesting genetic stability across host plants and geographic ranges. The moderate divergence from *P. zeae* (11.4 – 11.7 %) and *P. delattrei* (11.3 – 12.0 %) reaffirms the validity of *P. parazeae* as a distinct taxon. It reduces the risk of misidentification with other sympatric lesion nematodes commonly associated with rice in Southeast Asia.

The 18S rDNA region, though more conserved, further corroborated the identity and phylogenetic placement of *P. parazeae*, offering complementary support in multigene analyses. The congruence across two independent loci enhances confidence in species resolution and reflects best practices in nematode diagnostics (Subbotin *et al*., 2008).

Root-lesion nematodes are notorious for their capacity to cause chronic root damage, particularly under flooded or intermittently irrigated conditions common in Southeast Asian rice agroecosystems. The identification of *P. parazeae* from symptomatic rice roots in Vinh Phuc, Vietnam, raises concerns about its potential impact on crop health and yield in this region. Although pathogenicity assays were beyond the scope of this study, prior reports from China demonstrate that *P. parazeae* can be highly aggressive on sugarcane, leading to significant root necrosis and impaired nutrient uptake (Wang *et al*., 2015). Given the similar biology of lesion nematodes across host plants, it is plausible that *P. parazeae* may exhibit comparable virulence on rice, especially under stress conditions or in poorly managed soils.

This finding should prompt a reevaluation of nematode management protocols in Vietnamese rice systems. Current integrated pest management strategies for nematodes in rice often focus on *Hirschmanniella* spp. or *P. zeae* (Bridge *et al*., 2005; Sikora *et al*., 2018); the presence of *P. parazeae* adds complexity and necessitates updated diagnostic surveys, resistance screening, and possibly the development of targeted control measures, including crop rotation, organic amendments, and biological control agents. The discovery of *P. parazeae* on rice in Vietnam also contributes to our understanding of nematode dispersal and host adaptation. Its occurrence in diverse monocot hosts, including sugarcane, maize, and now rice, suggests a broad ecological amplitude and potential for host-switching within Poaceae. This adaptability may be facilitated by agricultural intensification, monoculture expansion, and soil disturbance, all of which create conducive environments for polyphagous endoparasitic nematodes (Bridge *et al*., 2005; Castillo & Vovlas, 2007).

Moreover, this report confirms that *P. parazeae* is not geographically restricted to southern China, but may be more widely distributed across subtropical and tropical Asia than previously recognized. The regional movement of infected planting materials, machinery, and water could facilitate further dissemination, emphasizing the need for phytosanitary monitoring and inclusion of *P. parazeae* in national and regional quarantine lists.
